# ‘For my own safety and peace of mind’: views on contraception among young emergency contraception users: a qualitative study in Swedish pharmacies

**DOI:** 10.1530/RAF-26-0085

**Published:** 2026-09-02

**Authors:** Minte ter Haar, Maral Shafei, Cecilia Arvidson, Anisa Assifi, Karin Emtell Iwarsson

**Affiliations:** ^1^ Department of Women’s and Children’s Health, Karolinska Institutet, Stockholm, Sweden; ^2^ Faculty of Medicine, Erasmus University Rotterdam, Rotterdam, The Netherlands; ^3^ Division of Gynaecology and Reproductive Medicine, WHO-Centre, Karolinska University Hospital Stockholm, Sweden; ^4^ SPHERE NHMRC Centre of Research Excellence, Department General Practice, School of Public Health and Preventive Medicine, Monash University, Melbourne, Victoria, Australia

**Keywords:** emergency contraception, contraception, young women, Swedish pharmacies, qualitative study

## Abstract

**Introduction:**

Despite well-established contraceptive care services in Sweden, there remains an unmet need for contraception. Emergency contraceptive pills are provided over the counter in pharmacies without subsequent contraceptive counselling. Use is widespread, yet research on Swedish emergency contraceptive users is limited. Understanding their experiences is important for tailoring contraceptive care to their needs.

**Objective:**

To explore young emergency contraceptive pill users’ experiences and perceptions on contraceptive decision-making and contraceptive use.

**Materials and methods:**

We performed a qualitative content analysis with an inductive approach on semi-structured interviews in 10 participants between 17 and 26 years old, who purchased emergency contraceptive pills in community pharmacies in Stockholm, Sweden.

**Results:**

The theme ‘Navigating reproductive autonomy through perceptions, information and norms’ was identified, supported by three categories: i) Emergency contraception at pharmacies facilitates reproductive autonomy, where participants shared their appreciation of easy access, ii) Internal perceptions and external information sources affect contraceptive use, considering factors such as one’s personal situation and social media, and iii) Perceived social norms and their role in contraceptive experiences, where influences from social context and perceived stigma were mentioned.

**Conclusion:**

To support informed decision-making and reproductive autonomy, providers need to ensure easy access to contraception, while sustaining non-judgemental environments and actively countering myths and misconceptions with accurate, evidence-based information both offline and online.

**Lay summary:**

This is a qualitative study of 10 interviews with participants aged 17–26 years, who purchased emergency contraceptive pills in community pharmacies in Stockholm, Sweden. We explored experiences and perceptions of these young women on contraceptive decision-making and contraceptive use. Our findings show that community pharmacies play a role in facilitating reproductive autonomy and providing emergency contraceptive pills with minimal barriers to access. Contraceptive decision-making is shaped by internal perceptions, external information sources and different perspectives through friends, family and societal norms. Young women’s views on contraception emphasise the wish for a personal approach suitable to their bodies and situational needs. They are navigating new information channels, in which social media plays a prominent role. Healthcare providers can play a role in creating a safe, supportive and non-judgemental environment, offline and online.

## Introduction

Contraception is a cornerstone of girls’ and young women’s health, first and foremost in preventing unintended pregnancy (Baird *et al.* 2025). Two nation-wide survey studies in Sweden from 2019 to 2022 showed that the unmet need for contraception increased from 15 to 17% and that the primary reason for contraceptive use among women was effective prevention of pregnancy (Hellstrom *et al.* 2019, Envall *et al.* 2022). The contraceptive decision-making process is a multifaceted process, shaped by individual, interpersonal and societal factors. Previous research has shifted the view of theories on contraceptive decision-making from short term to a decades-long journey subject to change (Simmons *et al.* 2023). A Swedish study on contraceptive use in early abortion-seeking women showed that over 60% of women used no contraception or a withdrawal method at the time of conception due to low fertility awareness, risking unintended pregnancy (Niemeyer Hultstrand *et al.* 2023).

Internationally, the availability of emergency contraceptive pills (ECPs) with or without prescription varies greatly. In Sweden, ECPs (which include levonorgestrel and ulipristal acetate) are widely available and accessible to women of all reproductive ages and are the only hormonal contraceptive option available without prescription (ECEC 2025). Since ECPs suppress or postpone ovulation, unprotected sex later in the same cycle after ECP use puts people at up to three times the risk of conceiving (Cameron *et al.* 2020), which emphasises the importance of easy access to regular contraception after ECP use.

Youth Clinics play a key role in the nationwide care for Swedish people between 12 and 25 years old. Their aim is low-threshold healthcare for adolescents and young adults, meaning it should be easy to seek the care they are entitled to (Föreningen för Sveriges ungdomsmottagningar 2016). We focus on young women, as they are provided with subsidised contraceptive care. In Sweden, contraceptive counselling is free of charge for individuals of all ages and contraception is free for those under 21 years of age and subsidised up to age 26 in the Stockholm Region, with a maximum cost of 100 SEK per year (approximately 10 euros).

Previous national studies on ECP users have described attitudes towards ECPs when ECPs were introduced over the counter at Swedish pharmacies; however, the findings are relatively dated (Häggström-Nordin & Tydén 2001, Aneblom *et al.* 2002, Gainer *et al.* 2003, Larsson *et al.* 2004, Ekstrand *et al.* 2013). To our knowledge, no recent study has explored young ECP users’ contraceptive perceptions in the context of community pharmacies in Sweden. Therefore, the aim of this study was to explore young ECP users’ experiences and perceptions on contraceptive decision-making and contraceptive use.

## Materials and methods

We employed a qualitative content analysis with an inductive approach. Our report follows the SRQR guidelines (O'Brien *et al.* 2014). The researchers’ reflexivity and positionality is reported in Supplementary Table 1 (see section on Supplementary materials given at the end of the article).

### Study setting and participant selection

This study was established for process evaluation as a sub-study of the LOWEC trial (Clinical Trials ID# NCT06299475). LOWEC is a cluster randomised crossover controlled trial conducted in 37 pharmacies in the Stockholm Region. The LOWEC trial examines whether composite intervention of online contraceptive counselling at the time of a pharmacy provision of ECPs, plus an invitation to a SRH clinic, will result in increased use of subsequent effective contraception (hormonal or intrauterine) compared with standard care. The online contraceptive counselling was modified from our previous trial on structured contraceptive counselling (Emtell Iwarsson *et al.* 2021).

Clients who bought ECPs over the counter at one of the included pharmacies were invited to participate. Eligibility criteria were women 15 years and older, visiting a pharmacy to purchase ECPs for her own use, with a smartphone with the ability to scan a QR code and with proficiency in Swedish or English, independently or with the help of an interpreter.

An online baseline questionnaire and follow-up questionnaires were collected at 1 and 12 months following the ECP provision for both intervention and control group participants. At baseline, eligible participants were asked whether they were interested in being interviewed. Those who responded ‘yes’ or ‘maybe’ were contacted via SMS or telephone after having completed the 1-month follow-up questionnaire, to schedule the interview. Participants received oral and written information about the study and had the possibility to ask questions before they signed the informed consent prior to the interviews. A purposive sampling strategy was applied to ensure variation in participants’ characteristics, including age, migration background, pharmacy chain and the area in which the pharmacy was located.

The findings from the randomised controlled LOWEC trial will be explored in a forthcoming publication.

### Data collection

Semi-structured individual interviews were conducted between October 2024 and April 2025. The concept of data saturation was discussed prior to the study and was continuously assessed during the interview process. Data collection continued until data saturation was reached and few new topics arose.

Interviews were conducted via Zoom or Teams in a quiet environment. The interview guide (Table 1) was adapted from the UK Bridge-it trial (Patterson *et al.* 2022). With pre-determined questions, the interviews explored thoughts on and experiences with previous, current and future regular and emergency contraception, social context and knowledge, influence and awareness. Questions were mainly open-ended, using probes to facilitate in-depth exploration. Relevant concepts that emerged from previous interviews were incorporated in subsequent interviews. Interviews were conducted with participants of all ages; however, this study exclusively focusses on interviews with participants aged 17–26 years. This focus was chosen because specific topics emerged during their interviews and young women are provided with subsidised contraceptive methods.

**Table 1:** Interview guide.

1. Would you like to tell me about when you bought emergency contraception (the morning-after pill)?
2. How did you experience being invited to participate in the study?
3. Would you like to share your previous experiences with contraceptives and emergency contraception?
4. Would you like to share your thoughts on contraceptives in general?
5. What are your thoughts on emergency contraception?
6. How do you feel your surroundings view emergency contraception and contraceptives?
7. When you went to buy emergency contraception – did you have any thoughts about contraceptives then? How did you feel about receiving contraceptive counselling?
8. Can you describe how you experienced the counselling material?
9. What did you think about the possibility of booking a contraceptive counselling appointment?
10. Can you tell me about your current use of contraceptives and your thoughts going forward?
11. How have you experienced participating in the study, and has it affected you in any particular way?
12. Do you have any thoughts on how this could be implemented in real life, beyond the study?
13. How did you experience this interview?

Regular contraceptive methods are defined as pills, patches, vaginal rings, injectables, implants and intrauterine devices (IUDs) and will be referred to as ‘contraceptive methods’ in this paper.

The term ‘woman’ is used for all persons who can get pregnant, regardless of their gender identity.

### Statistical analysis

All interviews were audio-recorded and transcribed verbatim in Swedish by the researchers (MS and CA) and a professional transcriber, including noteworthy non-verbal communicative elements, i.e. laughing and silences. Translation to English was performed (MtH) and checked by a native Swedish speaker (CA). Graneheim and Lundman’s inductive content analysis (Graneheim & Lundman 2004) was used to analyse the transcribed English data, performed manually by MtH. Meaning units were identified, condensed, decontextualised and coded, with codes kept close to the text, progressing from manifest to latent content. Codes were grouped and recontextualised to identify (sub)categories and a theme. All data were initially coded to ensure completeness. Non-relevant units were later excluded. Coding and categorisation were iteratively refined until a consensus was reached within the team. Transcripts were analysed with repeated revisiting of the data to ensure epistemological consistency.

### Ethical approval

Ethical clearance was approved by the Swedish Ethical Review Authority (Dnr 2023-06755-01 and amendment 2024-05936-02).

## Results

At baseline, 48 participants within the age range of 17–26 answered ‘yes’ or ‘maybe’ to being interviewed after the 1-month follow-up. A number of 18 participants were not contacted due to missing follow-up responses at 1 month (*n* = 6) or logistical reasons (*n* = 12). Thirty potential participants were contacted, of whom 20 could not be reached or declined interviews, resulting in 10 completed interviews. The mean length of the interviews was 35 min. The median age was 21.5 (range: 17–26). All participants had used ECPs containing levonorgestrel. Main contraceptive methods used were condoms or withdrawal at baseline and at the 1-month follow-up. Characteristics of the participants are listed in Table 2.

**Table 2:** Characteristics of participants (*n* = 10).

ID#	Age	At baseline		At 1 month		Previous	
		Main method(s)	Satisfaction level	Main method(s)	Satisfaction level	ECP use	Abortion
1	21	Pill	Very dissatisfied	Pill	Neither/nor	2–5	0
2	18	Condom	Neither/nor	Condom	Satisfied	2–5	0
3	23	Condom	Neither/nor	Pill	Satisfied	1	0*
4	23	Condom	Dissatisfied	Condom	Dissatisfied	1	0
5	21	Withdrawal	Satisfied	Withdrawal	Neither/nor	6–10	2
6	18	Condom, withdrawal	Dissatisfied	Condom, withdrawal	Dissatisfied	1	0
7	22	Condom, NFP	Satisfied	Condom, NFP	Satisfied	1	0
8	23	Condom, withdrawal	Dissatisfied	Withdrawal	Neither/nor	2–5	0
9	17	Condom	Satisfied	Condom	Satisfied	1	0
10	26	Withdrawal, NFP	Satisfied	Implant	Neither/nor	2–5	0

* Post-inclusion abortion.

NFP, natural family planning.

The content analysis process resulted in the overarching theme ‘Navigating reproductive autonomy through perceptions, information and norms'*.* This is built on three categories: i) Emergency contraception at pharmacies facilitates reproductive autonomy, ii) Internal perceptions and external information sources affect contraceptive use and iii) Perceived social norms and their role in contraceptive experiences. Furthermore, the categories are supported by subcategories as listed in Table 3.

**Table 3:** Overview of the participants’ responses analysed and presented in subcategories, categories, and a theme.

Subcategory	Category	Theme
Emergency contraception is a good option ‘in case’	Emergency contraception at pharmacies facilitates reproductive autonomy	Navigating reproductive autonomy through perceptions, information, and norms
Pharmacies are accessible for emergency contraception		
Taking control in decision making of emergency contraception use		
Safe places for education, guidance and provision		
Age in relation to the healthcare system		
Choosing contraceptive methods is weighing pros and cons	Internal perceptions and external information sources affect contraceptive use	
Need for regular contraception is situationally dependent		
Lack of feeling informed		
Online sources providing information		
Social media as a place for education and sharing experiences		
Influence of different perspectives and societal opinions	Perceived social norms and their role in contraceptive experiences	
Myths and misconceptions		
Embarrassment as an own conviction		
Regular contraception is more accepted than emergency contraception		

### Emergency contraception at pharmacies facilitates reproductive autonomy

This category covers access to and use of emergency contraception.

Unanimously, the availability of ECPs was perceived as positive, and participants appreciated its provision at pharmacies, which were considered accessible. ECPs were viewed as an important option, independent of participants’ views on hormonal contraception in general, but not as a sustainable method for repeated use. Participants’ reasons for ECP use included lack of contraception or contraceptive failure (e.g. missed birth control pill). All had previously used ECPs and reported reduced stress when using them again.

Despite ECPs being provided free of charge at Youth Clinics, participants did not find paying for ECPs at the pharmacy troublesome. Some expressed hesitation about visiting a Youth Clinic, as booking an appointment was seen as a ‘big step’ and a barrier for participants in accessing ECPs. One participant said she would not have used ECPs if it involved calling or booking an appointment, instead of immediate access at a pharmacy. Another explained she did not want to have any interaction with a healthcare provider (HCP) when getting ECPs, preferring the pharmacy over a Youth Clinic.

All but one participant had previous experience of visiting a Youth Clinic. Participants had visited Youth Clinics for SRH information and provision of regular and emergency contraception. In contrast to the before-mentioned hesitancy, Youth Clinics were generally seen as safe places to receive helpful advice and answers to questions regarding their reproductive health.

“That meant I didn’t know much about it. But I thought the information was really good, and I felt calm. I also got time – no one pressured me to start anything or insert anything or whatever. They just wanted to recommend things for my own safety and peace of mind.” ID9

A sense of urgency was the most prominent reason to get ECPs from a pharmacy. Youth Clinics’ limited opening hours or location was viewed as barriers, especially as some participants used ECPs as a precaution and reported feeling worried and anxious about the risk of pregnancy.

“And I’ve always bought my own emergency pills because I’ve been very paranoid, and I’ve felt like, ‘No, I need to take it now.’ And the Youth Clinic isn’t always open, and I don’t really go there much in general because it’s a bit out of the way. And yes, I know I could get it for free, but when I’m in one of those paranoid phases, I just want it right away. I’m not going to wait until the next day at 8 am.” ID5

The oldest participant, 26 years, provided additional insights into contraceptive behaviours and healthcare service provision at her age. Outside the Youth Clinic age range, she navigated her reproductive choices more autonomously, with a perceived reduction in access to low-threshold guidance.

“[…] it sometimes feels like they decide, maybe after 23 or after 25, that ‘now – you should already have done this. Now you should already have received all the counselling. Now you should have this figured out'.” ID10

A younger participant also provided insights into how age relates to perceived healthcare access. Although falling in the Youth Clinic age range, she was doubtful about where to go for contraceptive care:

“Now I don’t know. I’m 21 now, so I don’t know if I can still go to Youth Clinic, so then I actually don’t know where I would go. But I guess to a doctor, like a gynaecologist, or a midwife or something. Maybe?” ID1

### Internal perceptions and external information sources affect contraceptive use

This category covers the use of, information about and decision-making related to contraceptive methods. These are processes influenced by internal perceptions and external information sources.

Participants described the process of finding a suitable and satisfactory contraceptive method as weighing advantages and disadvantages. Many participants had tried multiple contraceptive methods, describing a process of experimentation, discontinuation and reassessment.

Differences in perceived convenience and practicality influenced participants’ contraceptive choices. Condoms were viewed as less reliable due to concerns about breakage, forgetting to use them and doubts about effectiveness. Daily adherence to the oral contraceptive pill was seen as challenging, with many participants reporting past inconsistent use or anticipating difficulty maintaining regular use. As a result, some preferred methods that offered continuous pregnancy protection, such as long-acting reversible contraception (i.e. implant or IUD). Contraceptives were also used for symptom management, such as reducing menstrual pain by suppressing bleeding.

Participants reported experiencing side effects from different contraceptive methods, such as deterioration in mental health, mood changes and other physical symptoms, which often led to method discontinuation. Irregular bleedings were seen as annoying and stressful and were another reason to stop using a method. The need for regular contraception was seen as situationally and age-dependent. All but one participant had a steady sexual partner. One participant elaborated on her preference for condoms when in a regular relationship:

“/…/ I have a few friends who use birth control pills, but now we’re at an age where we kind of claim – and believe – that we understand how a condom works. /…/ We don’t have that many different partners, so it’s not really relevant to have an implant or be on the pill or that kind of thing. Most of the people I know are in long-term relationships, so I think it’s very... it’s a bit more of a grown-up mindset now than when we were 16 and absolutely had to be on the pill just in case. But now we’re old enough that I think we understand what we’re doing and what the consequences are of certain things. So, we make decisions accordingly.” ID7

Participants expressed how external sources, such as school-based sexuality education and social media, affected their contraceptive use. Some participants expressed a shortcoming in sexuality education at a young age, which had focussed on menstruation, safe sex and consent. One participant shared that contraception was partially covered, leaving a knowledge gap she later resolved through her own initiative at a Youth Clinic. Participants appeared to have an open attitude towards learning about contraception. A perceived lack of information prompted many participants to express curiosity and wish to update their knowledge. We found that some participants felt contraception and method choice should not be approached by HCPs as a ‘quick fix*’* for young women, but as a personal, informed decision to protect themselves in the right way.

“I don’t know, maybe that there are better options, that I’m a bit uninformed. That maybe I need to read up a bit more on what the best option for me is.” ID4

All participants, except one, talked about the role of online information sources in providing reproductive health and contraception-related information. The role of social media in influencing participants’ views of contraception was evident through their different stories. Social media platforms, particularly TikTok and Instagram, were frequently, and independently of the interview guide, mentioned as key educational sources. Some participants, however, did raise doubts about the veracity of the information and noted the broad spectrum of sources.

“I think many people turn to TikTok or Instagram first. I’ve also noticed it’s become very popular to search or ask things like, ‘Did birth control pills work for you?’ There’s a kind of community there for women and young girls, where they educate each other. /…/ When it comes to contraception, everything is available online if needed.” ID5

“I’d say there are many different creators. Actually, before I came to this meeting, I saw a really sweet midwife who was talking about things like discharge. Someone had written a comment, and she responded /…/ Then there are regular creators who aren’t trained gynaecologists or midwives, but who talk about their experiences with things like the contraceptive implant or whatever /…/” ID5

Participants highlighted that social media has become a primary source of SRH information for themselves and their peers.

### Perceived social norms and their role in contraceptive experiences

This category covers the influence of different perspectives on contraceptive use.

Participants were aware of the opinions and perceptions on different contraceptive methods within their social network. Some shared how the oral contraceptive pill or IUD was common among friends, alongside both positive experiences (e.g. with the implant) and negative perceptions, such as friends who were *‘*against it*’* or had experienced painful IUD insertions. Mother’s or sisters’ experiences with a contraceptive method also influenced their contraceptive use. Views on sharing contraception use with parents were variable, with some keeping contraceptive use private, even when parents had expressed open attitudes. Overall, participants were exposed to differing views and social opinions on various contraceptive methods. While the direct influence on behaviour remains undetermined, perspectives within social networks appeared to shape participants’ own views. The influence was particularly evident for the oldest participant:

“/…/ the friends I had in high school didn’t talk that much about it beyond, ‘Are you on the pill?’ and then that was kind of it. Whereas my group of friends at university – it was very open to talk about everything. The ceiling is very high, and conversations can go very wide.” ID10

Some participants expressed fear or hesitancy towards trying contraceptive methods they did not have experience with. This fear stemmed from stories from their social network, online sources or social media. Anticipation of side effects focussed predominantly on the effect of hormones, with many participants expressing a strong aversion. Hormone hesitancy was commonly mentioned.

“I didn’t feel very comfortable taking them. It felt very chemical, almost. I know it’s just hormones, but it didn’t feel very natural to me.” ID6

There were concerns about contraception being dangerous or causing extreme side effects. Some participants said they were aware of it being hearsay; nevertheless, it caused fear or hesitancy towards contraception.

“A lot of people say different things about contraception and hormones. I’ve heard things like – now I know it’s not true – but like, ‘You can become sterile'.” ID2

In the abundance of online content, participants were exposed to a considerable amount of horror stories.

“/…/ I’ve heard so many horror stories, like ‘I couldn’t walk,’ ‘I almost died,’ and just a lot of stuff. And I know the sources are like TikTok and so on, but still, there’s been a lot about side effects. And that’s why I’ve been a bit hesitant. But now I’m like, well, you can at least try.” ID8

When exploring participants’ perceptions of contraception within a broader sociocultural context, Sweden was described as open and accepting. However, participants shared that ECPs carried some stigma and were perceived to be discussed less openly, relative to other contraceptive methods.

“I’d say it’s very accepting and encouraging in Swedish society, at least in the circles I’m in. It’s definitely not something people speak negatively about if you use contraception.

/…/ There’s a bit of a stigma when you go to get an emergency contraceptive pill. Like it’s a bit embarrassing, like you should feel a little ashamed. Uh, but I don’t know if that’s really how it is or if it’s just something you convince yourself of.” ID7

## Discussion

### Main findings

This study explored young ECP users’ experiences and perceptions of contraceptive decision-making and use. The overarching theme, ‘Navigating reproductive autonomy through perceptions, information, and norms', captures how decision-making is shaped by interacting individual, informational and social factors. Three key categories were identified: the role of community pharmacies in facilitating timely access to emergency contraception, the influence of internal perceptions and external information sources on contraceptive beliefs and use and the impact of social norms on contraceptive experiences. Together, these findings highlight contraceptive decision-making as a dynamic, multifaceted process.

Consistent with the existing literature, contraceptive decision-making should be viewed as a decades-long journey subject to change. Our findings align with previous research highlighting that contraceptive choice is shaped by a complex interplay of individual beliefs and knowledge, sociocultural context and health system factors, including perceived side effects and access to care (Simmons *et al.* 2023).

Our results underline the importance of community pharmacies for access to emergency contraception among young women. Despite the availability of free emergency contraception through Youth Clinics in Sweden, some young women still choose to turn to pharmacies, highlighting the importance of having multiple access points for ECPs. Ensuring availability across different healthcare settings is essential to meet diverse needs and preferences. Our findings further suggest that the reproductive autonomy of ECP users could be strengthened by clarifying where contraceptive counselling and contraceptives can be obtained, with an emphasis on the importance of collaboration between Youth Clinics and pharmacies.

Participants in our study expressed a preference for a method that provides a consistent sense of protection, yet described a hesitancy to start a new method, expressing fears towards side effects and a reluctance for hormonal methods. This is in line with previous results on attitudes towards contraception in Sweden (Hellstrom *et al.* 2019, Envall *et al.* 2022). While consultations with an HCP were seen as supportive in initial decision-making, discontinuation frequently occurred in response to side effects. Participants in our study seemed to rarely consult with their HCP for further advice on managing these side effects or exploring alternative contraceptive options. As a result, the risk of unintended pregnancy would reoccur. Side effects are known to be a leading cause for discontinuation, and there is evidence that comprehensive counselling on side effects and the possibility of method switching for users initiating new methods may be effective for improving continuation (Jain *et al.* 2019, Cavallaro *et al.* 2020). Structured follow-up by healthcare providers after contraceptive initiation could mitigate this issue by supporting method continuation, facilitating timely switching and reducing periods of non-use (Jain *et al.* 2019).

Information environments, particularly social media, played a central role in shaping contraceptive perceptions. Participants frequently accessed platforms such as TikTok and Instagram, often before consulting HCPs. While these platforms offer accessible and anonymous information, they also expose users to variable-quality content. Previous research consistently shows that contraception-related content on social media is dominated by personal experiences, often emphasising negative side effects and discontinuation, alongside misinformation and distrust of HCPs (Wu *et al.* 2023, Allen *et al.* 2025, de Moel-Mandel *et al.* 2025, Pfender *et al.* 2025). Pain during IUD insertion and distrust of HCPs was content often found on TikTok in three English-language studies (Wu *et al.* 2023, Allen *et al.* 2025, de Moel-Mandel *et al.* 2025). Our findings align with this evidence, with participants describing exposure to conflicting and sometimes unreliable information.

Despite this, HCPs remained important sources of information, particularly in final decision-making, consistent with findings from the European Parliamentary Forum for Sexual and Reproductive Rights (EPF 2024). This highlights an opportunity for HCPs to strengthen their role within digital health spaces. Content produced by HCPs, although less prevalent, has been shown to achieve higher engagement and credibility among young audiences (Pfender *et al.* 2025), suggesting that greater professional presence online may help counter misinformation.

Social norms and internalised stigma were found to shape participants’ experiences. Feelings of shame or embarrassment appeared to be linked to internalised beliefs, rather than perceived judgement from HCPs. These perceptions contributed to hesitancy in seeking care and reinforcing the use of online sources. Our results suggest a broader pattern in how young people seek information, turning to online sources that provide anonymity, perceived non-judgemental community and the ability to explore information at their own pace.

Interestingly, feelings of shame were more pronounced in relation to ECP use than to the use of a regular contraception. Some participants perceived regular contraception as a responsible choice, whereas ECP use was not associated with the same sense of responsibility. These findings underscore the importance of HCPs actively communicating and promoting a non-judgemental approach, which may help counteract internalised shame and encourage help-seeking behaviour.

We should not focus solely on making it easier for young women to engage in their reproductive health. They are taking the initiative, in ways, however, that are new and not fully integrated yet in the healthcare system. The challenge is how to respond to the effects of new information channels and changing views on contraception. A well-informed patient is essential for shared decision-making.

### Strengths and limitations

We have aspired to ensure trustworthiness throughout the initiation, execution and interpretation of this study and its data, following the criteria for credibility, transferability, dependability and confirmability as described by Lincoln & Guba (1985). Credibility of the findings is supported by our team’s collaborative approach, with interchangeable work roles and regular peer debriefing. Data collection and analysis were executed and checked by more than one researcher. All findings have been presented to multiple researchers along with original data to check consistency. We aimed to ensure transparency in team reflexivity and to report on all four criteria transparently in this paragraph and throughout the thesis.

A strength of this study is that the interviews were performed by experienced clinicians, which may have facilitated participants’ openness to take part and respond to the questions, resulting in rich and in-depth data. Furthermore, collaborating pharmacies covered the four largest Swedish pharmacy chains, and the 37 pharmacies were spread out in different municipalities in the Stockholm Region. Participants were also recruited from various municipalities, which altogether may enhance the transferability of these findings. Credibility was enhanced by a systematic and iterative analysis, including repeated readings of the transcripts and discussions among multiple analysts.

Limitations include a relatively older sample (median: 21.5), with an underrepresentation of the youngest participants. Younger participants were less likely to volunteer or cancelled their interview. Study materials were limited to Swedish and English, potentially excluding other groups. Interviews were conducted by a midwife and a gynaecologist, potentially introducing bias through participants’ interaction with an HCP. As participants were selected through voluntary interest, selection and volunteer bias may also have occurred.

## Conclusion

Community pharmacies play an important role in young women’s reproductive health, providing ECPs with minimal barriers to access. Our study highlights developments in Swedish young women’s perceptions and behaviours regarding reproductive health in recent years. Their views on contraception are emphasising the wish for a personal approach suitable to their bodies and situational needs. They are navigating new information channels, in which social media plays a prominent role. Reproductive autonomy of young women can be supported by creating a safe, supportive and non-judgemental environment, offline and online. HCPs can play a role in mitigating the narrative, providing patients with accurate, evidence-based information, and correct prevailing misconceptions about contraception and ECP use.

## Supplementary materials



## Declaration of interest

The authors declare that there is no conflict of interest that could be perceived as prejudicing the impartiality of the work reported.

## Funding

The LOWEC trial received funding from the European Society of Contraception and Reproductive Health (ESC) and from the Strategic Research Area Health Care Science (SFO-V) at Karolinska Institutet, Stockholm, Sweden.

## Author contribution statement

KEI and MS drafted the interview guide. CA and MS conducted the interviews. Content analysis was repeatedly performed by MtH, until a consensus was reached with all authors. MtH drafted the initial manuscript, and KEI, MS and AA significantly edited and critically reviewed the manuscript. All authors approved the final version of the manuscript.

## References

[bib1] Allen RH, Song S, Weir GM, et al. 2025 “Stop gaslighting your patients”: a quantitative and qualitative analysis of user experiences of IUDs on TikTok. J Pediatr Adolesc Gynecol 38 498–503. (10.1016/j.jpag.2025.02.003) 39938712

[bib2] Aneblom G, Larsson M, von Essen L, et al. 2002 Women’s voices about emergency contraceptive pills “over-the-counter”: a Swedish perspective. Contraception 66 339–343. (10.1016/s0010-7824(02)00367-0) 12443964

[bib3] Baird S, Choonara S, Azzopardi PS, et al. 2025 A call to action: the second lancet commission on adolescent health and wellbeing. Lancet 405 1945–2022. (10.1016/s0140-6736(25)00503-3) 40409329

[bib4] Cameron ST, Glasier A, McDaid L, et al. 2020 Use of effective contraception following provision of the progestogen-only pill for women presenting to community pharmacies for emergency contraception (Bridge-It): a pragmatic cluster-randomised crossover trial. Lancet 396 1585–1594. (10.1016/s0140-6736(20)31785-2) 33189179 PMC7661838

[bib5] Cavallaro FL, Benova L, Owolabi OO, et al. 2020 A systematic review of the effectiveness of counselling strategies for modern contraceptive methods: what works and what doesn’t? BMJ Sex Reprod Health 46 254–269. (10.1136/bmjsrh-2019-200377) PMC7569400 31826883

[bib7] de Moel-Mandel C, Donnelly A & Bugden M 2025 “Do you know what birth control actually does to your body?”: assessing contraceptive information on TikTok. Perspect Sex Reprod Health 55 358–367. (10.1111/psrh.70025) PMC12421087 40589123

[bib8] Ekstrand M, Tyden T, Darj E, et al. 2013 Twelve-month follow-up of advance provision of emergency contraception among teenage girls in Sweden-a randomized controlled trial. Upsala J Med Sci 118 271–275. (10.3109/03009734.2013.841308) 24102148 PMC4190889

[bib9] Emtell Iwarsson K, Envall N, Bizjak I, et al. 2021 Increasing uptake of long-acting reversible contraception with structured contraceptive counselling: cluster randomised controlled trial (the LOWE trial). BJOG 128 1546–1554. (10.1111/1471-0528.16754) 33988917

[bib10] Envall N, Wallstrom T, Gemzell Danielsson K, et al. 2022 Use of contraception and attitudes towards contraceptive use in Swedish women: an internet-based nationwide survey. Eur J Contracept Reprod Health Care 27 409–417. (10.1080/13625187.2022.2094911) 36004625

[bib11] European Consortium for Emergency Contraception 2025 Sweden – Emergency Contraception. Brussels: ECEC. (https://www.ec-ec.org/emergency-contraception-in-europe/country-by-country-information/sweden/)

[bib12] European Parliamentary Forum for Sexual and Reproductive Rights 2024 Youth Contraceptive Awareness Study. (https://www.epfweb.org/node/1113)

[bib13] Föreningen för Sveriges ungdomsmottagningar 2016 Guidelines for Swedish Youth Centres, 1st edn. Stockholm: FSUM. (https://fsum.nu/wp-content/uploads/2022/03/fsum-guidelines.pdf)

[bib14] Gainer E, Blum J, Toverud EL, et al. 2003 Bringing emergency contraception over the counter: experiences of nonprescription users in France, Norway, Sweden and Portugal. Contraception 68 117–124. (10.1016/s0010-7824(03)00114-8) 12954524

[bib15] Graneheim UH & Lundman B 2004 Qualitative content analysis in nursing research: concepts, procedures and measures to achieve trustworthiness. Nurse Educ Today 24 105–112. (10.1016/j.nedt.2003.10.001) 14769454

[bib16] Hellstrom A, Gemzell Danielsson K & Kopp KH 2019 Trends in use and attitudes towards contraception in Sweden: results of a nationwide survey. Eur J Contracept Reprod Health Care 24 154–160. (10.1080/13625187.2019.1581163) 30920325

[bib17] Häggström-Nordin E & Tydén T 2001 Swedish teenagers’ attitudes toward the emergency contraceptive pill. J Adolesc Health 28 313–318. (10.1016/s1054-139x(00)00164-6) 11287250

[bib18] Jain A, Aruldas K, Tobey E, et al. 2019 Adding a question about method switching to the method information index is a better predictor of contraceptive continuation. Glob Health Sci Pract 7 289–299. (10.9745/ghsp-d-19-00028) 31249024 PMC6641810

[bib19] Larsson M, Eurenius K, Westerling R, et al. 2004 Emergency contraceptive pills over-the-counter: a population-based survey of young Swedish women. Contraception 69 309–315. (10.1016/j.contraception.2003.11.013) 15033406

[bib6] Lincoln YS & Guba EG 1985 Naturalistic Inquiry. Sage Publications.

[bib20] Niemeyer Hultstrand J, Törnroos E, Tydén T, et al. 2023 Contraceptive use among women seeking an early induced abortion in Sweden. Acta Obstet Gynecol Scand 102 1496–1504. (10.1111/aogs.14630) 37493190 PMC10577618

[bib21] O'Brien BC, Harris IB, Beckman TJ, et al. 2014 Standards for reporting qualitative research: a synthesis of recommendations. Acad Med 89 1245–1251. (10.1097/acm.0000000000000388) 24979285

[bib22] Patterson S, McDaid L, Saunders K, et al. 2022 Improving effective contraception uptake through provision of bridging contraception within community pharmacies: findings from the Bridge-it study process evaluation. BMJ Open 12 e057348. (10.1136/bmjopen-2021-057348) PMC8845311 35149574

[bib23] Pfender EJ, Tsiandoulas K, Morain SR, et al. 2025 Hormonal contraceptive side effects and nonhormonal alternatives on Tiktok: a content analysis. Health Promot Pract 26 407–411. (10.1177/15248399231221163) 38166482

[bib24] Simmons RG, Baayd J, Waters M, et al. 2023 Assessing contraceptive use as a continuum: outcomes of a qualitative assessment of the contraceptive journey. Reprod Health 20 33. (10.1186/s12978-023-01573-4) 36793112 PMC9930211

[bib25] Wu J, Trahair E, Happ M, et al. 2023 TikTok, #IUD, and user experience with intrauterine devices reported on social media. Obstet Gynecol 141 215–217. (10.1097/aog.0000000000005027) 36473194 PMC9892286

