# Supplementary material for: ‘For my own safety and peace of mind’: views on contraception among young emergency contraception users: a qualitative study in Swedish pharmacies
Source: Reprod Fertil. 2026 Sep 2;7(3):RAF260085. doi: 10.1530/RAF-26-0085 (PMC13548106; doi:10.1530/RAF-26-0085)
Supplement: Supplementary file 1 [file RAF-26-0085_supplementary_table_1.pdf]

Supplementary Table 1. Researchers' characteristics and reflexivity

MtH is a recently certified medical doctor from Rotterdam, The Netherlands. She performed this research when she was a Master student in Medicine in the context of a Master's thesis and on a voluntary basis, subsequently.<br><br>KEI is a postdoctoral researcher in contraception and contraceptive counselling, with a particular research focus on migrants and young people. She is a registered nurse-midwife with extensive clinical experience from Youth Clinics in Stockholm, Sweden, where she continues to work part-time.<br><br>MS is a medical doctor and PhD student in reproductive health. The LOWEC trial forms part of her doctoral research, and she conducted approximately half of the interviews. Through her clinical training in Obstetrics and Gynecology, she has experience of contraceptive counseling and reproductive healthcare from her clinical work at a university hospital in Stockholm, Sweden. The relatively small age difference between MS and some participants may have facilitated rapport and mutual understanding during interviews.<br><br>CA is a research assistant in contraception and contraceptive counselling and a retired nurse-midwife. She is a former manager of a Youth Clinic and has extensive clinical experience from Youth Clinics, working with and meeting young people, in Stockholm, Sweden. She conducted half of the interviews.<br><br>During the interviews MS and CA remained mindful that their professional backgrounds could influence both the interaction and the interpretation of participants' accounts.

AA is a research fellow (AA) in Melbourne, Australia, conducting research in adolescent sexual and reproductive health (SRH) and in the pharmacy setting in Australia and internationally.

No prior relationships existed between researchers and participants. To support reflexivity, the analysis was conducted by a multidisciplinary research team with diverse professional backgrounds, including researchers without experience of Youth Clinics, researchers of different ages, and researchers from different countries. Interpretations were discussed and critically examined throughout the analytical process to enhance the credibility of the findings.
